# Understanding pandemic experience of older South Asians in Chinese society in Hong Kong during COVID-19

**DOI:** 10.1093/geroni/igaf122.805

**Published:** 2025-12-31

**Authors:** Zhijing Qu, Daniel W L Lai, Jiajia Zhou

**Affiliations:** Hong Kong Baptist University, Kowloon, Hong Kong, China (People’s Republic); Hong Kong Baptist University, Kowloon, China (People’s Republic); Hong Kong Baptist University, Kowloon, Hong Kong, China (People’s Republic)

## Abstract

The COVID-19 pandemic significantly disrupted lives worldwide, yet the experiences of marginalized groups, particularly older individuals in racialized minority communities, have received limited attention. This study aims to explore the social contexts of older South Asians in Hong Kong during the pandemic. Through in-depth narrative interviews with 20 South Asians aged 65 and over, thematic analysis revealed the critical roles of social capital in bonding, bridging, and linking. Participants highlighted the importance of family and close friends in bonding social capital, providing emotional, informational, and social support. Bridging social capital was evident in interactions with neighbours and the local Chinese community, who offered support but could also be sources of discrimination. Linking social capital was demonstrated through the provision of essential resources and services by local, voluntary, and religious organizations. These findings underscore the multifaceted functions of social capital in promoting social inclusion and support for aging racialized minorities, especially during health crises. The study highlights the urgent need for targeted policies and interventions to address the unique challenges faced by older South Asians in Hong Kong. By understanding the intricate dynamics of social capital within these communities, policymakers can more effectively tailor efforts to build robust and inclusive support systems, fostering a more resilient and equitable society in the face of future pandemics or similar crises.

